# Neurotrophins and spinal circuit function

**DOI:** 10.3389/fncir.2014.00059

**Published:** 2014-06-05

**Authors:** Vanessa S. Boyce, Lorne M. Mendell

**Affiliations:** Department of Neurobiology and Behavior, Stony Brook UniversityStony Brook, NY, USA

**Keywords:** neurotrophin, spinal cord injury, motor neuron, stretch reflex, pain, *c-Fos*, locomotion, nociceptor

## Abstract

Work early in the last century emphasized the stereotyped activity of spinal circuits based on studies of reflexes. However, the last several decades have focused on the plasticity of these spinal circuits. These considerations began with studies of the effects of monoamines on descending and reflex circuits. In recent years new classes of compounds called growth factors that are found in peripheral nerves and the spinal cord have been shown to affect circuit behavior in the spinal cord. In this review we will focus on the effects of neurotrophins, particularly nerve growth factor (NGF), brain derived neurotrophic factor (BDNF) and neurotrophin-3 (NT-3), on spinal circuits. We also discuss evidence that these molecules can modify functions including nociceptive behavior, motor reflexes and stepping behavior. Since these substances and their receptors are normally present in the spinal cord, they could potentially be useful in improving function in disease states and after injury. Here we review recent findings relevant to these translational issues.

## Introduction

Classical studies of spinal cord function have centered on well-defined neural pathways that were considered to mediate stereotyped automatic functions such as stretch and flexion reflexes, and to distribute input from sensory fibers and from descending fibers to their appropriate targets. In the past several decades it has become apparent that these projections are modifiable and that this plays an important role in the physiology and pathology of functions mediated by the spinal cord. Thus a complete understanding the nature of spinal function as well as spinal dysfunction requires knowledge of these processes. In addition, such plasticity can serve potentially as a basis for reversing pathological changes after injury or disease. Initial studies of this type centered on the effects of monoamines on spinal circuits because of the well-known projections of serotonergic and noradrenergic projections to the spinal cord from the raphe and locus coeruleus, respectively (Jankowska et al., 2000). More recently, a different class of molecule has been shown capable of altering spinal circuit function. These are the neurotrophins, the most studied of which are nerve growth factor (NGF), brain derived neurotrophic factor (BDNF), and neurotrophin-3 (NT-3). These molecules interact with neurons that express the appropriate tropomyosin related kinase (trk) receptor- trkA (NGF), trkB (BDNF), and trkC (NT-3) as well as the p75 receptor common to all trk- expressing neurons. The receptor biology and intracellular signaling associated with trk receptors is reviewed elsewhere (Chao and Hempstead, 1995; Huang and Reichardt, 2001).

## Neurotrophin effects on sensory neurons and their spinal projections

### NGF

NGF was the first neurotrophin to be well characterized after discovery of its ability to promote the growth of neurites when applied to explanted dorsal root ganglia (reviewed in Cowan, 2001). Later, it was identified as a survival factor during the period of programmed cell death for small sensory neurons expressing the trkA receptor (reviewed in Mendell, 1995; Mendell et al., 1999b; Huang and Reichardt, 2001). In the immediate postnatal period, NGF continues to act as a survival factor, and subjecting the animal to an anti NGF antibody leads to failure of high threshold mechanoreceptors to survive (Lewin et al., 1992). Beginning about 2 days after birth, withdrawing NGF has little effect on nociceptor survival. However, during this same postnatal period, these cells begin to show sensitization of the response to noxious heat or capsaicin after application of NGF (Zhu et al., 2004). This switch, occurring in the immediate postnatal period, is associated with upregulation of ERK1/2 and PI3K/P110 in dorsal root ganglion (DRG) cells, and does not appear to be due to changes in expression of trkA or TRPV1 (Zhu and Oxford, 2011).

The sensitization elicited by NGF in adult nociceptive neurons in the DRG results from an increase in the current produced by TRPV1 receptor activation (Shu and Mendell, 1999a,b; Galoyan et al., 2003). This increase is not due to enhanced sensitivity of the TRPV1 receptors, but rather from recruitment of additional TRPV1 receptors from the interior of the cell (Zhang et al., 2005; Stein et al., 2006). The ability of NGF to sensitize the response of nociceptors to noxious heat stimuli has important behavioral significance, namely that exposure of adult rats to systemic NGF results in robust mechanical and thermal hyperalgesia. Thermal hyperalgesia has a strong peripheral component while mechanical hyperalgesia induced by exposure to NGF is mediated largely centrally (Lewin et al., 1994). The central effects elicited from peripheral exposure to NGF are mediated by another neurotrophin, BDNF, whose expression in the DRG is enhanced several hours after administration of NGF (reviewed in Pezet and McMahon, 2006). The synaptic action of BDNF is discussed in more detail below.

### NT-3

NT-3 plays a role similar to NGF in the survival of sensory afferents, but in this case it is muscle spindle (group Ia) afferent fibers expressing trkC (reviewed in Huang and Reichardt, 2001). NT-3 also promotes the elaboration of their terminal projections to motor neurons during late stages of development (Chen et al., 2003), and in addition potentiates the strength of the group Ia synaptic projection to motor neurons. In intact afferents this has been studied by delivering the NT-3 systemically or intramuscularly (Arvanian et al., 2003; Petruska et al., 2010) and it is difficult to determine the precise mode of action. For peripherally axotomized afferent fibers the NT-3 was delivered via a cuff on the cut nerve (Mendell et al., 1999). Because the effects were limited to the treated nerve, it seems likely that this was due to a direct effect of the NT-3 on the terminals of the fibers that were exposed to the NT-3, but possible mechanisms such as sprouting or facilitation of glutamate release could not be distinguished.

The monosynaptic projections from spindle afferent fibers to motor neurons also exhibit potentiation acutely when exposed to NT-3 in the isolated spinal cord (Arvanov et al., 2000). The acute effect is developmentally regulated because the postsynaptic (motor neuron) NMDA receptors required for the action of NT-3 become inactive about 10–14 days after birth due to Mg block (Arvanian and Mendell, 2001a; Arvanian et al., 2004). NT-3 also enhances the mechanical sensitivity of the neuroma of spindle afferents that have been axotomized and capped with a Gore-Tex sleeve (Munson et al., 1999).

### BDNF

The effect of the trkB agonist BDNF on sensory neurons is more complex. Although BDNF also serves as a survival factor for sensory neurons during the period of programmed cell death, the correspondence to a particular phenotype as has been observed with NGF (nociceptors) and NT-3 (proprioceptors) is not clearcut. Furthermore, another neurotrophin, NT-4, also binds with the trkB receptor and can substitute for BDNF in some, but not in all cases, to assure the survival of sensory neuron populations (e.g., Erickson et al., 1996). BDNF, like NGF and NT-3, is known to regulate the sensitivity of low threshold sensory receptors, in this case the slowly adapting Type I mechanoreceptor (Carroll et al., 1998); NT-4 regulates the sensitivity of a different low threshold mechanoreceptor, the D- Hair (Hilaire et al., 2012). Thermal nociceptors are also reported to be sensitized by NT4 (Rueff and Mendell, 1996) and by BDNF (Shu et al., 1999; Shu and Mendell, 1999b).

An additional property of BDNF is that its expression in nociceptor somata is upregulated by exposure to NGF in the periphery as part of the response to peripheral inflammation (Pezet and McMahon, 2006). Enhanced expression of BDNF in the DRG results in increased levels of BDNF in the superficial dorsal horn (Lever et al., 2001). This is known to rapidly enhance the synaptic action of nociceptive afferents on cells of lamina II via a mechanism involving NMDA receptors (Garraway et al., 2003). It is interesting that NMDA receptors in these cells are not subject to Mg block in the adult unlike those in motor neurons (see above), presumably because their subunit composition differs (Arvanian et al., 2004).

BDNF also acutely strengthens the monosynaptic reflex pathway in the isolated spinal cord of neonates. However, this reverses into long lasting inhibition of transmission several minutes after BDNF exposure (Arvanian and Mendell, 2001b). The inhibitory effect is thought to be due to a presynaptic effect of BDNF in contrast to the potentiation, which has a postsynaptic mechanism, as is the case for NT-3 (reviewed in Mendell and Arvanian, 2002). In agreement with these long lasting postnatal changes, exposure of the prenatal rat to BDNF by i.p. injection of the mother reduces the monosynaptic EPSP measured postnatally, whereas prenatal exposure to NT-3 increases it (Seebach et al., 1999).

### Summary

In summary, there are several common features linking the effects of the different neurotrophins on sensory neurons. All sensory neurons express trk receptors whose identity determines which neurotrophin will influence their function. Neurotrophins all serve as survival factors during the period of programmed cell death. They all affect the response of receptors to natural stimulation, and finally they all are able to modify the synaptic effects of the affected sensory neurons. In the latter case the mechanisms are quite different. NGF elicits its effect by upregulating another neurotrophin, BDNF, which elicits its effect in the spinal cord by acting on NMDA receptors in cells of the superficial dorsal horn. The effect of peripherally delivered NT-3 is not established, but probably involves a presynaptic change in the affected fibers. However, studies of the acute action of NT-3 have also revealed the possibility of a postsynaptic NMDA receptor- mediated change as discussed for BDNF.

## Neurotrophin effects on circuits in the injured spinal cord

Since spinal neurons differ in their relationship with the inputs and outputs, we evaluate the spinal effects of neurotrophins by investigating their action on functional circuits rather than on individual neurons or neuron classes. In some cases the circuit is sensory and is driven by specific sensory receptors. An example is a circuit that processes nociceptive input. Another circuit is the pattern generator which delivers an output causing stepping. A third circuit is the sensorimotor circuit responsible for the stretch reflex that converts muscle stretch into muscle contraction.

Although NGF has played a prominent role in investigating the role of neurotrophins on sensory fibers, most studies of neurotrophin action on spinal circuit function have been limited to BDNF and NT-3. TrkB and trkC receptors are expressed at a higher level than trkA receptors within the spinal cord (Liebl et al., 2001) thus raising the expectation that BDNF and NT-3 would be more effective in affecting spinal circuit function. Here we discuss the effect of BDNF and NT-3 on three circuits based on their effects on three behaviors: stepping, nociception, and the stretch reflex.

### Stepping

Application of neurotrophins to the injured spinal cord at the time of thoracic transection promotes stepping behavior in both cats (Boyce et al., 2007) and rats (Jakeman et al., 1998; Blits et al., 2003; Boyce et al., 2012b). Boyce et al. (2012b) reported that rats with intraspinal administration of AAV viruses engineered to express BDNF to a site just caudal to the T10 transection site could carry out plantar stepping with their hind limbs on a treadmill. Remarkably, these rats could also carry out overground stepping across a stationary surface requiring the provision of balance support only (i.e., without treadmill assistance). Training was not required for BDNF to exert any of these effects which developed over the first 2–4 weeks after transection and exposure to the AAV/BDNF.

Further evidence for BDNF- activation of neurons involved in stepping was a finding of elevated expression of *c-Fos* in cells of the intermediate zone in the upper lumbar spinal cord of these rats. Relatively little increase of *c-Fos* expression was observed in the ventral horn. There was a rostrocaudal gradient of *c-Fos* expression after administration at T10 such that it was greater at L2 than at L4/L5. Expression of *c-Fos* was not associated with enhanced neural activity *per se* because the rats had been anesthetized for an electrophysiological experiment for several hours prior to sacrifice. It is more likely that its expression was a direct effect of BDNF on these cells, as has been observed previously in dissociated embryonic hippocampal cells (Paul et al., 2001). However, *c-Fos* expression provided an indication that BDNF influenced L2 interneurons. Since L2 intermediate zone interneurons are likely crucial participants in the central pattern generator (CPG; reviewed in Goulding, 2009), it seems likely that this increase in *c-Fos* expression reflects the action of interneurons whose activity is responsible for the stepping behavior elicited by BDNF, i.e., *c-Fos* is a biomarker for stepping, at least under these conditions. The mechanism of BDNF action on this circuits remains to be determined, i.e., whether it is a generalized increase in excitability, activation of a specific subgroup of cells in this circuit or of cells driving it.

These findings in the transected cord suggest that the exogenous BDNF acts on trkB receptors to elevate activity in neurons of the locomotor circuit. This effect may be diminished to some extent by the presence of truncated trkB receptors whose expression is elevated in the spinal cord after contusion (Liebl et al., 2001). These authors suggest that the truncated receptors with a non-signaling intracellular component act to sequester BDNF thereby reducing its effect. More recent data have shown that deleting the truncated form of trkB receptor improves locomotor recovery after spinal contusion injury even without provision of exogenous BDNF (Wu et al., 2013).

### Nociception

Elevation of BDNF levels also results in increased sensitivity to a noxious heat stimulation as would be expected by sensitizing or activating cells in the superficial dorsal horn (Garraway et al., 2003). Cells in this region express elevated *c-Fos* indicating a response to BDNF as reported for cells in the intermediate zone. These findings raise the question as to whether activating nociceptive circuits is a requirement for BDNF to elicit overground stepping, i.e., stepping is a component of the response to noxious stimulation. There are no direct studies of this issue, but a recent report (Garraway et al., 2011) indicates that the relationship between BDNF, stepping and nociception is at the very least quite complex.

In experiments comparing the effects of NT-3 and BDNF on stepping under comparable conditions, NT-3 promoted only treadmill-assisted stepping with the further requirement, not needed for BDNF, for perineal stimulation accomplished by squeezing the base of the tail (Boyce et al., 2012b). Furthermore, NT-3 did not sensitize the response to nociceptive stimulation. Consistent with both of these findings was the finding of no increase in *c-Fos* expression in the spinal cord, either in the intermediate zone or in the superficial dorsal horn. It is not known whether this is due to differences in signaling elicited by trkB and trkC activation or whether there are few trkC receptors on cells eliciting the nociceptive and stepping behavior.

Another model demonstrating the pro-nociceptive role of BDNF comes from the response to peripheral nerve injury. Under these conditions BDNF has been shown to be released from microglia in the superficial dorsal horn that have been sensitized by the purinergic transmitter ATP released from the central terminals of damaged nociceptors (Tsuda et al., 2003). The activated microglia release BDNF that in turn reduces the level of the Cl^−^ transporter KCC2 in neurons of the dorsal horn. This diminishes the inhibitory action of GABA, and in extreme cases converts it to an excitatory action (Coull et al., 2005). Thus neurotrophins can be released from non-neuronal cells, a mechanism that has been known for many years in the periphery where Schwann Cells associated with damaged peripheral fibers release neurotrophins whose identity is associated with the fiber type (reviewed in Lehmann and Höke, 2010).

### Motoneurons and the monosynaptic reflex

BDNF causes increased excitability of motor neurons in the injured cord as evidenced by a reduced rheobase (Gonzalez and Collins, 1997; Boyce et al., 2012b) despite the lack of significantly altered *c-Fos* expression in the ventral horn. Of interest in this context is the finding that BDNF delivered in a single dose 24 h after transection has been reported to increase expression of the KCC2 chloride transporter in motor neurons of transected preparations (Boulenguez et al., 2010). This would oppose any intrinsic increase in excitability of motor neurons by enhancing the magnitude of inhibition due to establishment of a more negative chloride equilibrium potential. However, chronically transected preparations treated with continuous BDNF administration via engineered AAV viruses exhibit reduction in expression of the KCC2 transporter (see also preliminary data in Boyce et al., 2012a; Ziemlinska et al., 2014). This is an important area that requires further study, particularly in view of the recent finding indicating elevated levels of GABA and GABA- synthetic enzymes caudal to spinal transection (Ziemlinska et al., 2014). A combined reduction in motor neuron rheobase and a reduction in synaptic inhibition of motor neurons would be expected to promote spasticity as has been reported after BDNF administration (Boyce et al., 2012b; Fouad et al., 2013; Ziemlinska et al., 2014).

The effect of NT-3 on motor neuron excitability is opposite to that of BDNF, i.e., a reduced excitability (increased rheobase; see also Petruska et al., 2010; Boyce et al., 2012b). However, the decrease in intrinsic motor neuron excitability after NT-3 treatment is opposed by an increase in monosynaptic EPSP amplitude and a decrease in the after hyperpolarization (AHP) following the spike, each of which should enhance the strength of excitatory response directly (elevated EPSP) or indirectly (diminished AHP). Unlike NT-3, BDNF has no effect on the strength of synaptic projections to motor neurons in these preparations although as discussed above it enhances motor neuron excitability and can reduce synaptic inhibition via reduced KCC2 at least under some conditions.

### Selectivity of neurotrophin expression

**Figure 1:**
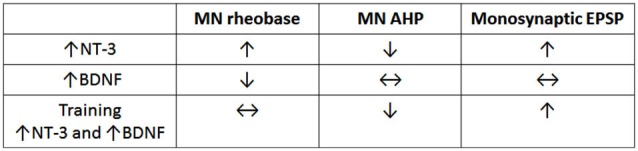
**Comparison of effects of NT-3, BDNF and step training on electrophysiological properties of motor neurons**. Note that training gives results that are qualitatively equivalent to the sum of the separate effects of NT-3 (data row 1) and BDNF (data row 2). The data are derived from Petruska et al. (2007), Petruska et al. (2010) and Boyce et al. (2012b).

Figure 1 shows that *summing* the separate effects of BDNF and NT-3 on the electrophysiology of motor neurons and their spindle synaptic input gives a result that is in qualitative agreement with the effects of motor training in spinal transected rats (Petruska et al., 2007) where the levels of BDNF and NT-3 are both increased (Ying et al., 2005; Côté et al., 2011; de Leon et al., 2011). This is consistent with the idea that training exerts its effects via changes in neurotrophin expression (Gómez-Pinilla et al., 2001). The increase in both BDNF and NT-3 after motor training differs from what is observed after increase in activity limited to proprioceptive pathways where NT-3 levels are increased but BDNF levels are not (Gajewska-Wozniak et al., 2013). This indicates that locomotor training involves more than altering the stretch reflex pathway despite its enhancement after step training (Petruska et al., 2007), and the beneficial effects of increasing it by operant conditioning (Thompson et al., 2013). These beneficial effects of stretch reflex enhancement may be due to improved weight support which facilitates stepping elicited by independent activation of the CPG.

The pathway- selective increase in neurotrophin levels is also observed for BDNF in that inflammatory pain results in upregulation of BDNF in trkA- expressing nociceptive neurons in the DRG (Pezet and McMahon, 2006) with subsequent release into the superficial dorsal horn of the spinal cord (Lever et al., 2001). Thus activity- driven elevation in neurotrophin levels is circuit- dependent and acts to potentiate the central effect of the activated fibers.

## Neurotrophins and repair of spinal circuits

Investigating a possible role for neurotrophins in the treatment of spinal injuries was prompted by earlier results in the developing spinal cord suggesting that these agents could encourage growth of axons. Thus there are many studies in which bridges of Schwann cells, olfactory ensheathing glia or other substrates were treated with neurotrophins to enhance elongation of cut axons (reviewed in Boyce et al., 2014; Boyce and Mendell, 2014). Additional studies in which neurotrophins were applied either to the severed proximal end of descending fibers in the vicinity of a spinal injury or to the cell body in the brainstem or cerebral cortex revealed their ability to promote the growth of axons descending from more rostral centers with some specificity. For example, BDNF stimulates growth of vestibulospinal fibers whereas NT-3 enhances corticospinal fiber growth although the specificity of action may not be absolute (see reviews in Boyce et al., 2014; Boyce and Mendell, 2014). As expected from the specificity of neurotrophin receptor expression on sensory axons (see Section Neurotrophin Effects on Sensory Neurons and their Spinal Projections), NGF enhances the ability of nociceptive afferent fibers to grow past a crush injury in the dorsal root and terminate in the appropriate area of the superficial dorsal horn whereas after a similar injury NT-3 stimulates growth of proprioceptors into the ventral horn (see review by Smith et al., 2012). At present, there is little information concerning direct effects of neurotrophins on the activity of spinal neurons recorded *in vivo*, in part because any observed activity changes might be an indirect action, e.g., due to activation of sensory fibers. Nonetheless, direct effects on spinal neuron activity would be expected because there are reports of trk receptors on these neurons (Liebl et al., 2001). Furthermore, some of the synaptic effects of exogenously applied neurotrophins in the isolated spinal cord have been attributed to trk receptors on the postsynaptic spinal neurons (Arvanov et al., 2000; Garraway et al., 2003).

Long term intrathecal treatment with neurotrophins can also promote the development of novel functional pathways in the spinal cord, specifically the formation of functional detours around zones of injury both in neonatal (Arvanian et al., 2006) and adult rats (García-Alías et al., 2011; Schnell et al., 2011). In adults the neurotrophin works best if supplemented with agents that overcome the inhibitory effect of the tissue on axonal growth, e.g., anti Nogo or chondroitinase (reviewed in Fawcett et al., 2012), and provision of NMDA subunit NR2D to bring these receptors back to their juvenile state (Arvanian et al., 2004). In these experiments, neurotrophins were introduced intrathecally using either engineered fibroblasts or viruses. The fibroblasts or the cells infected by the AAV viral vectors released the neurotrophin which interacted with the trk or p75- (reviewed in Nicol and Vasko, 2007) expressing cells resident in the CNS. Some success has also been reported in altering connections to motoneurons retrogradely by administering viruses expressing a neurotrophin to muscle (Fortun et al., 2009; Petruska et al., 2010). The peripheral administration approach might have important translational advantages.

Many investigators are now making efforts to repair the injured spinal cord using cell transplants including stem cells. In evaluating the results of these procedures it is important to keep in mind that these cells constitutively release neurotrophins (Lu et al., 2003), which could affect the function of spinal neurons and circuits in addition potentially promoting axonal elongation.

## Maladaptive effects of neurotrophins

Although neurotrophins have numerous potentially useful effects on the injured spinal cord, there are many potential drawbacks. Improving stepping might be negated by increased pain or spasticity both of which have been reported (Boyce et al., 2012b; Fouad et al., 2013; see review by Weishaupt et al., 2012). This may be the result of the multiplicity of actions of neurotrophins, particularly BDNF which elicits activity- based plasticity as well as activating microglia leading to pain. One might question why the same molecule has developed adaptive and maladaptive roles. In evaluating this issue it is important to remember that the different mechanisms are being studied in isolation in preparations that are often so severely injured (e.g., peripheral nerve injury, spinal cord injury) that any evolutionary significance is doubtful.

It remains to be seen whether adjusting the dosage of BDNF will help to balance the positive and negative effects of this treatment so that it might be used translationally. Alternatively, an approach allowing BDNF release to be turned on and off using a regulatable viral system might be possible. Finally, a discrete infusion of BDNF into motor pools via an indwelling catheter might improve the balance between the positive and negative effects of this agent. In support of this approach, Mantilla et al. (2013) recently demonstrated enhanced respiration leading to increased survival of cervically hemisected rats after chronic infusion of BDNF into the phrenic motor neuron pool.

## Conclusion

**Figure 2:**
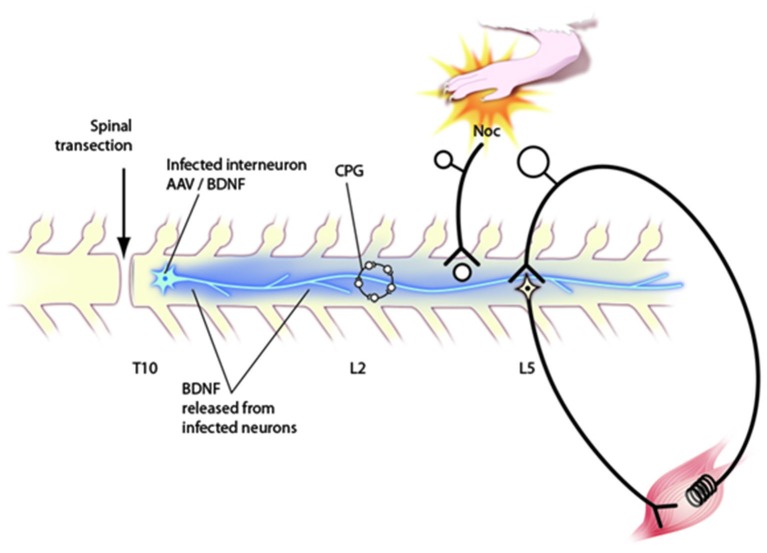
**Schematic diagram of circuitry affected by neurotrophins after application via engineered AAV viruses to the transected spinal cord**. Behavioral, electrophysiological and immunocytochemical data indicate that such injections affect the CPG in upper lumbar segments, nociceptive processing in the dorsal horn and the stretch reflex pathway. Further discussion of these effects in the text.

It appears, then, that neurotrophins, particularly BDNF, can have numerous actions that affect circuit function in the spinal cord (Figure 2). It follows that any attempt to use neurotrophins to promote repair will require targeting them to specific classes of cells, e.g., the CPG but not the nociceptive circuits in the superficial dorsal horn. So far this has not been possible using engineered cells, viral vectors or osmotic minipumps. Alternatively, it may be possible to devise cell based treatments which are easily turned off to control side effects. At present, what is clear is that neurotrophins are powerful molecules that could be very useful in repairing the injured cord. It will be necessary to recognize that they have significant effects on functional properties of cells and circuits as well as on axon elongation and to take this into account when devising protocols for their use.

## Conflict of interest statement

The authors declare that the research was conducted in the absence of any commercial or financial relationships that could be construed as a potential conflict of interest.
